# Magnetic Resonance Imaging-Proton Density Fat Fraction vs. Transient Elastography-Controlled Attenuation Parameter in Diagnosing Non-alcoholic Fatty Liver Disease in Children and Adolescents: A Meta-Analysis of Diagnostic Accuracy

**DOI:** 10.3389/fped.2021.784221

**Published:** 2022-01-11

**Authors:** Shuangzhen Jia, Yuzhen Zhao, Jiaqi Liu, Xu Guo, Moxian Chen, Shaoming Zhou, Jianli Zhou

**Affiliations:** Division of Gastroenterology, Shenzhen Children's Hospital, Shenzhen, China

**Keywords:** non-alcoholic fatty liver disease, children, diagnosis, controlled attenuation parameter, magnetic resonance imaging, meta-analysis

## Abstract

**Background and Aim:** Non-alcoholic fatty liver disease (NAFLD) is the most common cause of chronic liver disease in children and adolescents, and its prevalence increases with obesity. Magnetic resonance imaging (MRI) and transient elastography (TE) have been widely used to non-invasively evaluate NAFLD in adults. This study aimed to determine the efficacy and accuracy of MRI-proton density fat fraction (MRI-PDFF) and TE-controlled attenuation parameter (TE-CAP) in distinguishing hepatic steatosis in children and adolescents.

**Materials and Methods:** In this meta-analysis, the PubMed, Cochrane Library, Embase, Medline, and Web of Science databases were searched for articles that reported studies on the accuracy of MRI-PDFF or TE-CAP in grading the steatosis in children and adolescents with NAFLD. This study compared the sensitivity, specificity, and hierarchical summary receiver operating characteristic curves (HSROCs) of MRI-PDFF and TE-CAP in distinguishing between steatosis grades S0 and S1–3.

**Results:** A total of eight articles involving 874 children and adolescents with NAFLD were included in this study. The proportions of steatosis grades were 5 and 95% for S0 and S1–3, respectively. MRI-PDFF accurately diagnosed S1–3 steatosis, with a summary sensitivity of 0.95 (95% CI, 0.92–0.97), specificity of 0.92 (95% CI, 0.77–0.98), and HSROC of 0.96 (95% CI, 0.94–0.98). Likewise, TE-CAP accurately diagnosed S1–3 steatosis, with a summary sensitivity of 0.86 (95% CI, 0.70–0.94), specificity of 0.88 (95% CI, 0.71–0.96), and HSROC of 0.94 (95% CI, 0.91–0.95). Following a “positive” measurement (over the threshold value) for S1–3, the corresponding post-test probabilities of MRI-PDFF and TE-CAP for the presence of steatosis reached 92 and 88%, respectively, at the pretest probability of 50%. When the values were below the mentioned threshold values (“negative” results), the post-test probabilities of MRI-PDFF and TE-CAP became 5 and 13%, respectively.

**Conclusion:** Both MRI-PDFF and TE-CAP are highly accurate non-invasive methods to grade the hepatic steatosis in children and adolescents with NAFLD. Furthermore, MRI-PDFF is significantly more accurate in assessing steatosis grade than TE-CAP.

**Systematic Review Registration:** PROSPERO, identifier: CRD42021220422.

## Introduction

Non-alcoholic fatty liver disease (NAFLD) is the main cause of chronic liver disease in children and adolescents and the second most common cause of liver transplantation (1). Worldwide, ~2.6–11.3% of children and adolescents, and ~40–70% of obese children and adolescents have been reported to be diagnosed with NAFLD (1). Moreover, it is the leading cause of liver disease among children in China (2, 3). Hepatic steatosis (HS) refers to an essential histological hallmark of NAFLD. It represents the first stage of hepatic disease and results from the accumulation of excessive intrahepatocellular fat (4). Accordingly, HS should be accurately evaluated for early detection of NAFLD, whereby the prognosis can be improved and an effective treatment course can be determined.

The gold standard for diagnosing NAFLD is liver biopsy. However, this procedure is invasive and limited by sample size and hence easily leads to misdiagnosis (5). Steatosis grade is based on the standardized histological NAFLD scoring system (proportion of hepatocytes containing fat macrovesicles; grade 0 for <5%, grade 1 for 5–33%, grade 2 for 33–66%, and grade 3 for >66%) (6). It is difficult for children to endure the pain resulting from the biopsy; thus, this procedure is generally applied to adults and only exceptionally to children (7).

Recently, a non-invasive examination method called controlled attenuation parameter (CAP) has been developed by using transient elastography (TE) and adopted to evaluate HS (8). CAP can detect >5% of HS and accurately distinguish mild HS from moderate and severe hepatic steatoses. The CAP value increases with the fat content (9) and can be directly measured using FibroScan without being subjectively affected by the operator. It critically impacts the screening and diagnosis of fatty liver disease; the epidemiological investigation, follow-up, and monitoring of chronic liver disease; and the evaluation of liver transplantation (8, 10). However, the existing data regarding CAP have largely been derived from adults with chronic liver disease (11, 12). There are only a few reports on CAP-based evaluation of NAFLD in children and adolescents (13).

It has been proposed that magnetic resonance imaging (MRI) can be used as an alternative approach to TE-CAP (2). MRI-proton density fat fraction (MRI-PDFF) uses a gradient echo sequence with a low flip angle to minimize the T1 bias (2). This technique acquires multiple echoes, with the fat and water signals nominally in phase or out of phase relative to each other. Subsequently, the data obtained at the respective echo are passed to a fitting algorithm that estimates and corrects the T2 effects. The fat signals are modeled, the fat and water proton densities are measured, and the fat content is thereby calculated (14). It has been reported that MRI-PDFF accurately classifies grades and variations in HS, with a sensitivity of 80.0 to −95.8% and specificity of 83.6–100% (15, 16). Quantitative estimation of liver fat can be achieved using magnetic resonance spectroscopy (MRS) according to the current guidelines.

Several studies have shown that HS in adults can be highly accurately evaluated using TE-CAP and MRI-PDFF (2, 13, 16, 17). However, only a few studies have reported the use of TE-CAP in diagnosing NAFLD in children and adolescents. Moreover, the data generated in adults cannot be directly applied to children (18) because children differ from adults in several key aspects, such as body habitus, breath-hold capacity, and ability to tolerate imaging examinations. These factors can affect the feasibility, quality, and technical optimization of imaging examinations and their diagnostic performance (2, 19). Therefore, this study aimed to evaluate the accuracy of MRI-PDFF and TE-CAP in diagnosing NAFLD of children and adolescents.

## Methods

### Search Strategy and Selection Criteria for the Meta-Analysis

The diagnostic accuracy of the two techniques was assessed *via* a meta-analysis. Toward this end, we systematically searched the PubMed, Cochrane Library, Embase, Medline, and Web of Science databases for studies that assessed the accuracy of MRI-PDFF or TE-CAP in diagnosing NAFLD in children and adolescents. The studies included in our analysis had been published prior to March 17, 2021.

The search terms included were as follows: children, adolescents, teenagers and youths, pediatric^*^ non-alcoholic fatty liver disease, non-alcoholic fatty liver disease^*^, non-alcoholic fatty liver disease^*^, non-alcoholic fatty liver^*^, non-alcoholic steatohepatitis^*^, NAFLD, magnetic resonance^*^, MRI, transient elastography^*^, controlled attenuation parameter, TE, FibroScan, CAP, diagnostic accuracy, and diagnostic test accuracy. The reference lists of the retrieved articles were also searched manually.

This meta-analysis was conducted by complying with the preferred reporting items for the systematic review and the meta-analysis of diagnostic test accuracy articles (PRISMA-DTA) guidelines (20). The criteria for the inclusion of articles were the following: (a) the article evaluated the accuracy of MRI-PDFF or TE-CAP, in comparison with histological assessment, MRS-PDFF, or any other detection method as the reference, in diagnosing the HS in children and adolescents with NAFLD (aged 6–18 years); (b) the article provided sufficient information to construct a 2 × 2 contingency table and reported the rates of true and false positives and negatives; (c) the article included ≥20 patients to obtain good reliability; and (d) the article was published in English. For articles with overlapping patient samples, only the study with the larger cohort was included. The exclusion criteria were the following: (a) articles that did not use MRI or TE for evaluating the HS in NAFLD; (b) articles that used MRI or TE but did not study fatty liver; (c) articles that did not provide concrete data to calculate the diagnostic performances of MRI and TE; (d) articles that reported studies in animals; and (e) articles that did not report original or sufficient data.

### Procedures

Two investigators (Liao and Shi) independently extracted the data, including those related to quality assessment from the retrieved articles. Different opinions were resolved by the investigators in a consensus discussion. A third investigator (Yang) resolved discrepancies where an agreement could not be reached. The information of the patients [e.g., the total number of patients, age, body mass index [BMI], and the number of patients in each stage of HS] and the performance index of each diagnostic method [e.g., sensitivity, cutoff value, specificity, true positive and negative values, false positive and negative values, and receiver operating characteristic (ROC) value] were extracted from the respective articles included in this study. Other extracted variables were the publication year, first author, population, reference test, and region. The Quality Assessment of Diagnostic Accuracy Articles (QUADAS-2) score was used to evaluate the quality of the included articles (21).

### Statistical Analysis

The sensitivity, specificity, positive predictive value, net present value, hierarchical summary ROC (HSROC) curve, and positive and negative likelihood ratios were analyzed using Review Manager 5.4.1. The Stata version 15.0 was used for Fagan diagram analysis. This study also adopted an exact binomial rendition of the bivariate mixed-effects regression model (developed by van Houwelingen) for the meta-analysis of the treatment trials, modified for the synthesis of diagnostic test data. This model does not transform pairs of sensitivity and specificity of individual articles into a single indicator of diagnostic accuracy but preserves the two-dimensional nature of the data by considering the accuracy and correction between the two.

Based on the mentioned model, this study estimated the mean logit sensitivity and specificity with their standard errors and 95% CIs, the between-study variability in logit sensitivity and specificity, and the covariance between the two. The mentioned quantities were back-transformed to the original ROC scale to obtain the specificity, summary sensitivity, and diagnostic odds ratios. The derived logit estimates of sensitivity, specificity, and respective variances were then adopted to construct a hierarchical summary ROC for MRI-PDFF and TE-CAP with the summary operating points for the sensitivity and specificity on the curves, and with a 95% confidence contour ellipsoid (two-dimensional CI).

Heterogeneity was assessed by calculating the *I*^2^ value. The study-specific covariate publication bias was also adopted. Moreover, this study constructed the effective sample size funnel plots vs. the log diagnostic odds ratio and conducted a regression test of asymmetry.

The MIDAS module was employed for the STATA (version 15) software to conduct the bivariate summary ROC analysis. In this study, graphs were produced using the MIDAS module and the Quality Assessment of Diagnostic Accuracy Studies module for STATA.

### Assessment of Heterogeneity and Publication Bias

A random-effects model was used to analyze the influencing factors. The *Q*-test and the *I*^2^ statistic were performed to evaluate the heterogeneity among the studies. Finally, Deeks' funnel plot asymmetry test was performed to investigate the publication bias.

### Role of the Funding Source

This work was supported by the Science Technology and Innovation Committee of Shenzhen (2021N062-JCYJ20210324115408023).

## Results

### Search Results and Study Characteristics

**Figure 1:**
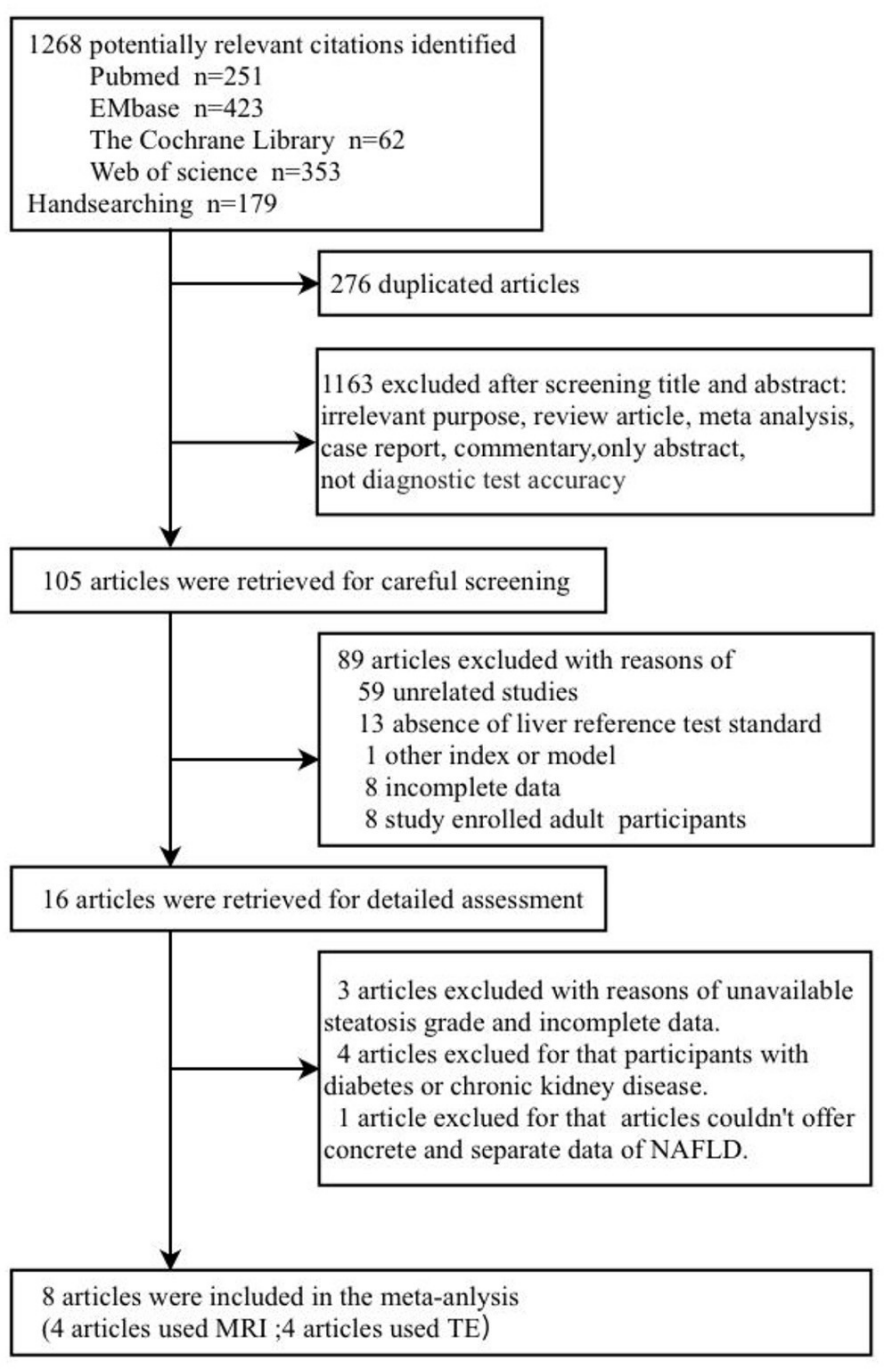
The articles included in the meta-analysis.

A total of 1,268 published articles were retrieved from the databases and used in this study. After screening of the articles and removal of the irrelevant ones, eight articles (9, 19, 22–27) were included in this study (Figure 1). It was found that four articles used MRI-PDFF (24–27), and four used TE-CAP (9, 19, 22, 23). Table 1 presents a list of the main features of the articles included in this study. It was found that all the articles reported that the radiologists were blinded to the liver histology results. Furthermore, the included articles had good methodological qualities (Table 1) as indicated by the QUADAS-2 scale.

**Table 1:** Characteristics of the included pediatric studies, comparing imaging-based evaluation of steatosis with a quantitative reference diagnostic test.

**Reference, region**	**Study/center description**	**Population**	**Number**	**Age range or mean age, y**	**Interval between the reference test and MRI/ TE**	**BMI (kg/m^**2**^) or BMI *z*-score**	**Blinded**	**Comparator**	**Definition of fatty liver**	**Steatosis stage (S0/S1–S3)**
**MRI-PDFF**										
Martino et al. (26), Italy	Prospective, single-center	Children	27	8–18 y	Within 14 d	28.7 ± 4.1 (kg/m^2^)	Yes	Histology	> 5% of hepatocytes with steatosis	0/27
Schwimmer et al. (25), USA	Prospective, single-center	Children	174	18–17 y	Within 100 d	33.3 ± 6.6 (kg/m^2^)	Yes	Histology	> 5% of hepatocytes with steatosis	24/150
Tang et al. (24), USA	Prospective, single-center	Children	77	8–18 y	Within 180 d	2.3 ± 0.4 (score)	Yes	Histology	> 5% of hepatocytes with steatosis	5/72
Zhao et al. (27), CHN	Prospective, single-center	Children	65	9–17 y	Within 90 d	26.3 ± 5.3 (kg/m^2^)	Yes	Spectroscopy	MRS-PDFF > 5%	25/40
**TE-CAP**										
Desai et al. (19), Italy	Prospective, single-center	Children	69	16.03 ± 2.9 y	Within 40 d	22.6 (kg/m^2^)	Yes	Histology	> 5% of hepatocytes with steatosis	46/23
Runge. et al. (23), Netherlands	Prospective, single-center	Children	60	8–18 y	Within 30 d	> 2(score)	Yes	Spectroscopy	MRS-PDFF > 4.14%	24/36
Shin. et al. (22), USA	Prospective, single-center	Children	86	7–18 y	Within 19 d	26.3 ± 4.9(kg/m^2^)	Yes	MRI	MRI-PDFF > 6%	10/76
Ferraioli. et al. (9), Italy	Prospective, single-center	Children	289	8–18 y	Within 30 d	26.8 ± 4.1(kg/m^2^)	Yes	Ultrasound	The ultrasonic test is positive	182/107

*BMI, body mass index; MRI, magnetic resonance imaging; TE, transient elastography; MRI-PDFF, magnetic resonance imaging-proton density fat fraction; TE-CAP, transient elastography-controlled attenuation parameter*.

### Diagnostic Performances of MRI-PDFF and TE-CAP in Assessing HS

**Figure 2:**
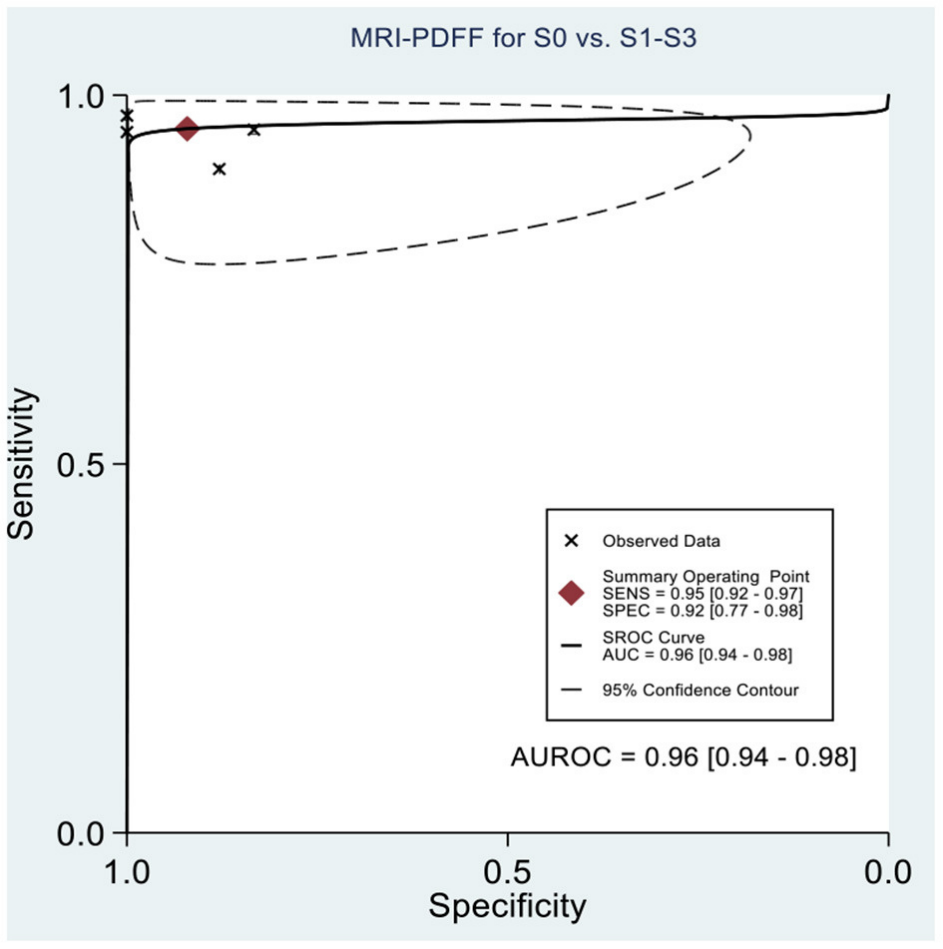
Hierarchical summary receiver operating characteristic (HSROC) curves for the detection of hepatic steatosis (S0 vs. S1–S3) by using MRI-PDFF or TE-CAP.

**Figure 3:**
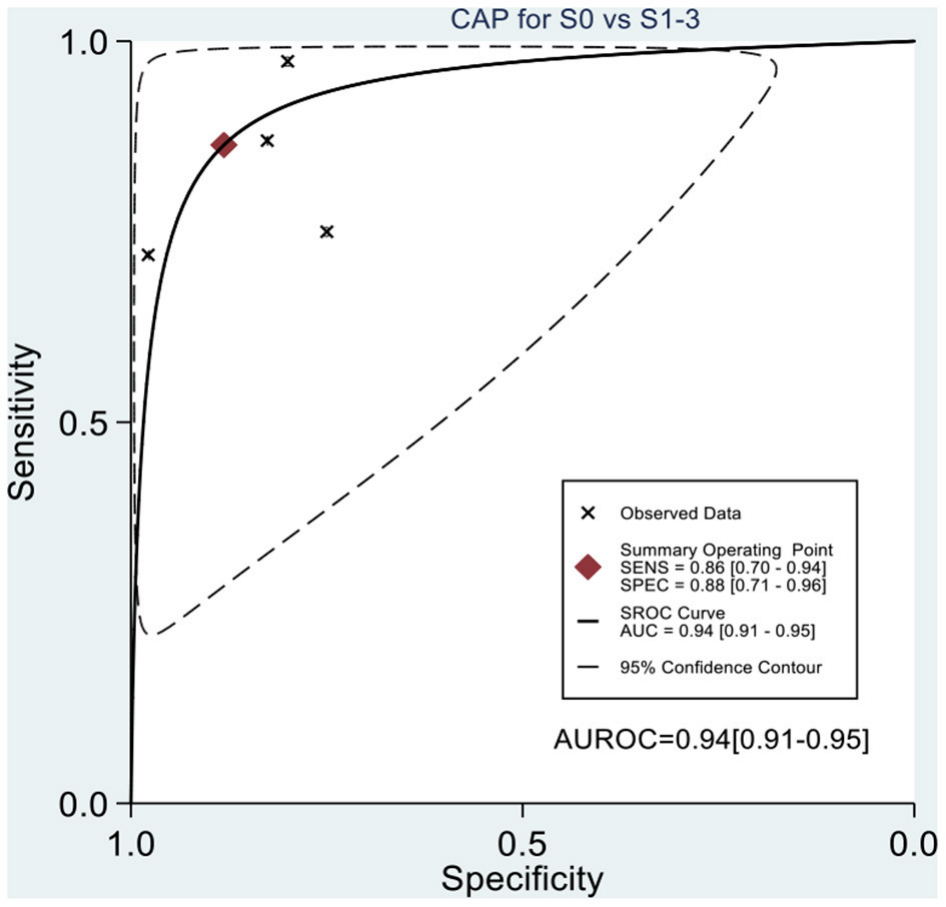
Hierarchical summary receiver operating characteristic (HSROC) curves for the detection of hepatic steatosis (S0 vs. S1–S3) by using MRI-PDFF or TE-CAP.

Table 2 shows the diagnostic performances of MRI-PDFF and TE-CAP for steatosis grading based on HSROC analyses. It was found that the MRI-PDFF exhibited high sensitivity and specificity for S1–3, with a high HSROC. The results of this study indicate that MRI-PDFF achieved the diagnostic accuracy for the detection at stage 1, with a summary sensitivity of 0.95 (95% CI, 0.92–0.97), specificity of 0.92 (95% CI, 0.77–0.98), and HSROC of 0.96 (95% CI, 0.94–0.98) (Figure 2). CAP achieved the diagnostic accuracy for the detection of steatosis stage S1–3, with a summary sensitivity of 0.86 (95% CI, 0.70–0.94), specificity of 0.88 (95% CI, 0.71–0.96), and HSROC of 0.94 (95% CI, 0.91–0.95) (Figure 3). Thus, compared with TE-CAP, MRI-PDFF exhibited better diagnostic performance.

**Table 2:** Study characteristics.

**Reference, region**	**TP**	**FP**	**TN**	**FN**	**Sensitivity%**	**Specificity%**	**Cutoff values**	**AUC**
Martino et al. (26), Italy	18	4	29	2	89	88	3.5%	–
Schwimmer et al. (25), USA	143	4	20	7	95	83	3.5%	0.9
Tang et al. (24), USA	70	0	5	2	97	100	6.4%	0.989
Zhao et al. (27), CHN	38	0	25	2	95	100	5.1%	0.991
Desai et al. (19), Italy	20	8	38	3	87	83	225 dB/m	0.93
Shin et al. (22), USA	74	2	8	2	98	80	241 dB/m	0.941
Runge et al. (23), Netherlands	27	6	18	9	75	75	277 dB/m	0.8
Ferraioli et al. (9), Italy	77	4	178	30	72	98	251 dB/m	0.84

*TP, true positive; FP, false positive; TN, true negative; FN, false negative*.

### Clinical Application of MRI-PDFF and TE-CAP for Assessing HS

**Figure 4:**
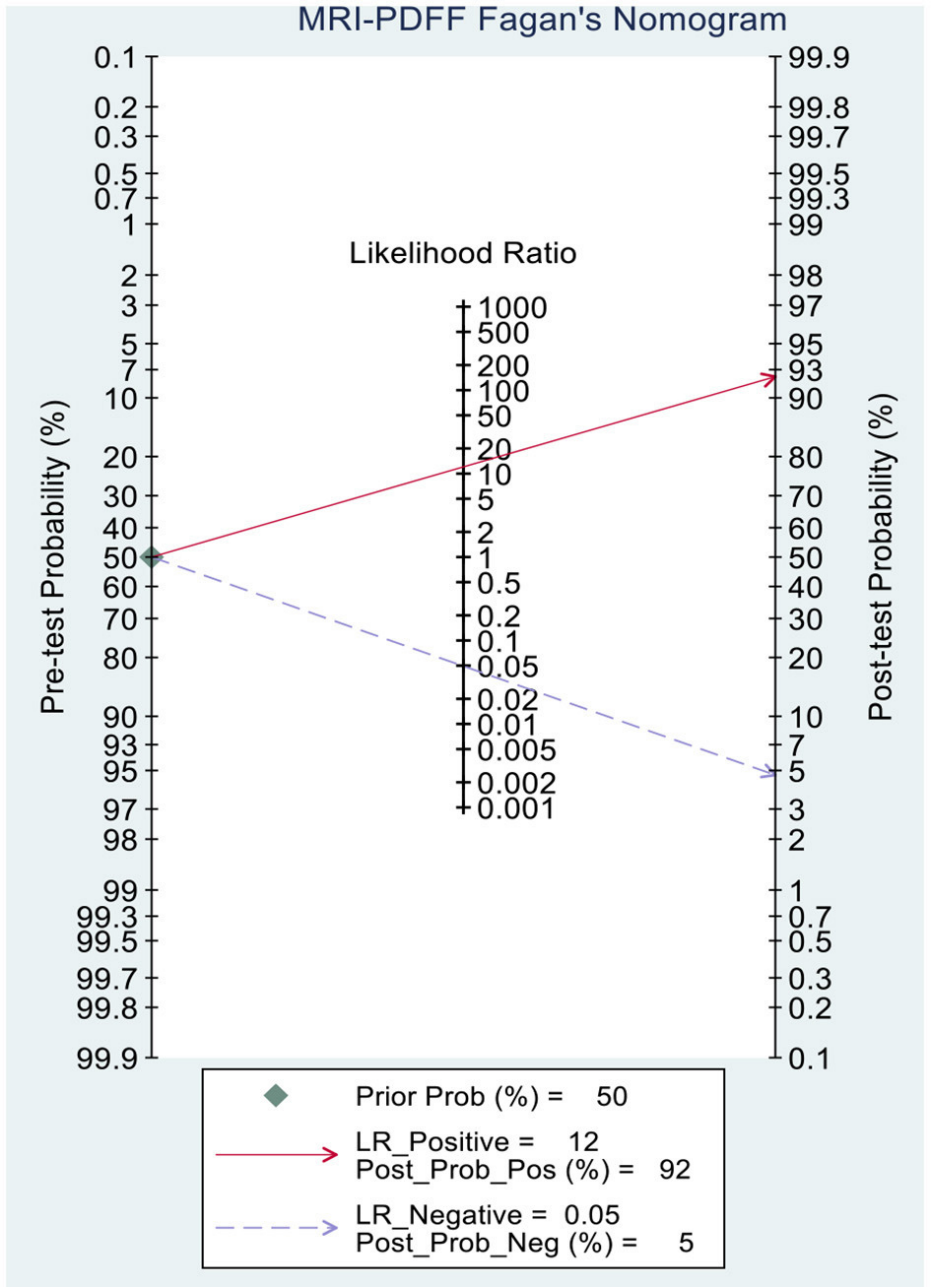
Fagan plot analysis to evaluate the clinical utility of MRI-PDFF and TE-CAP (S0 vs. S1–S3).

**Figure 5:**
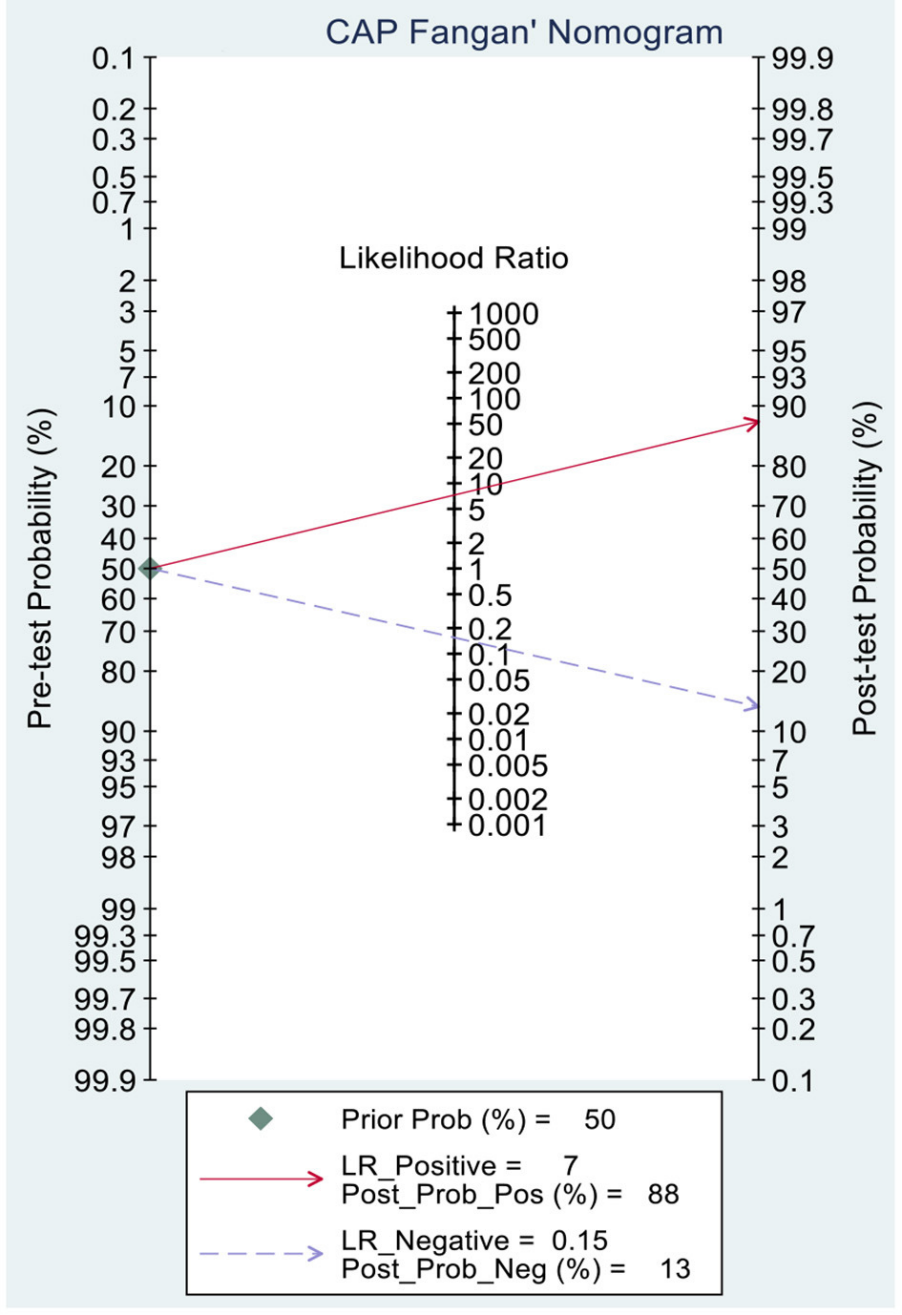
Fagan plot analysis to evaluate the clinical utility of MRI-PDFF and TE-CAP (S0 vs. S1–S3).

This study evaluated the pre-test probabilities of 50% vs. the corresponding post-test probabilities. The Fagan plot analysis demonstrated that MRI-PDFF achieved a 92% probability of correctly detecting S1–3 following a “positive” result when the pre-test probability was 50%. It was also found that the diagnosis would be wrong with a “negative” measurement in only 5% of the patients (Figure 4). Furthermore, the Fagan plot analysis demonstrated that TE-CAP was informative, with an 88% probability of correctly detecting S1–3 following a “positive” TE-CAP result at the pre-test probability of 50%. In addition, the diagnosis would be wrong with a “negative” measurement in 13% of the patients with a pre-test probability of 50% (Figure 5). It was also evident that MRI-PDFF has a better performance in detecting HS than TE-CAP.

### Publication Bias and Sensitivity Analyses

**Figure 6:**
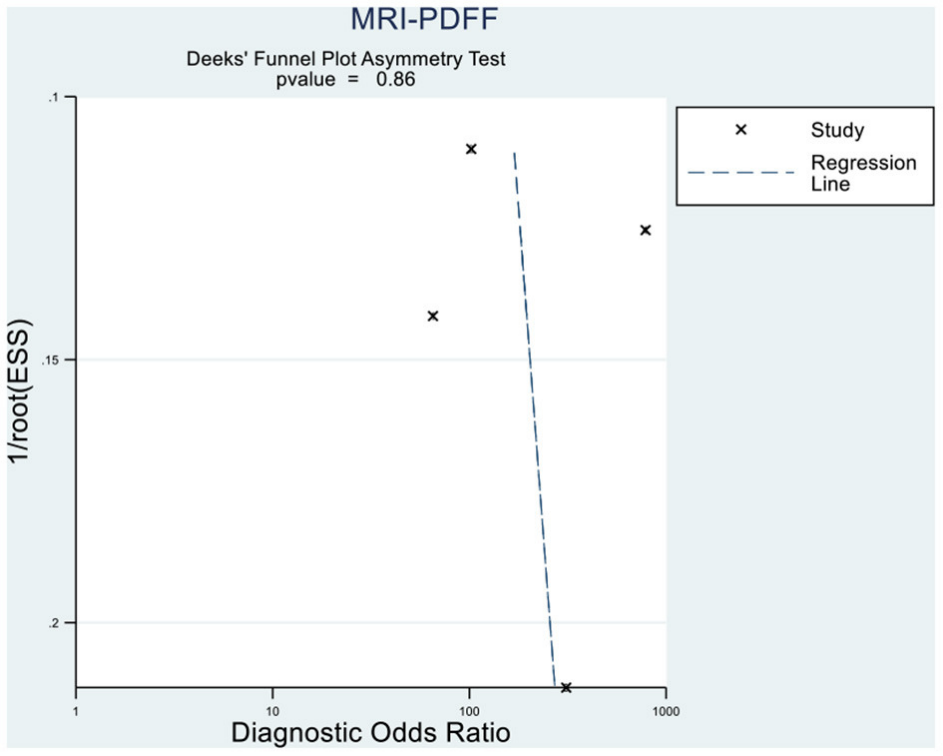
Deeks' funnel plot asymmetry test to evaluate the publication bias of MRI-PDFF and TE-CAP (S0 vs. S1–S3).

**Figure 7:**
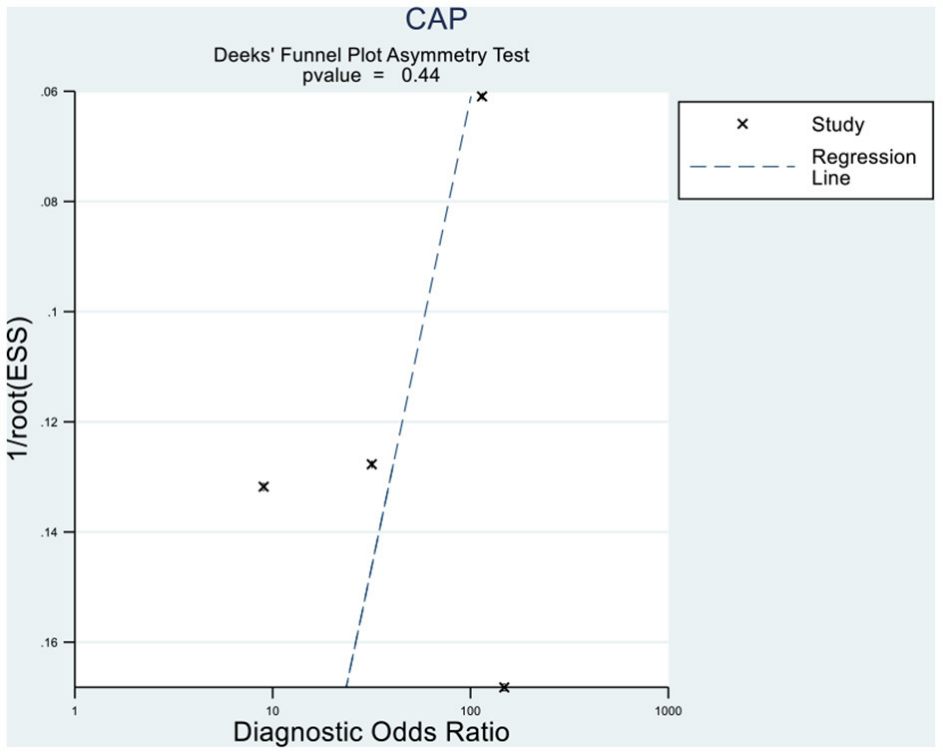
Deeks' funnel plot asymmetry test to evaluate the publication bias of MRI-PDFF and TE-CAP (S0 vs. S1–S3).

**Figure 8:**
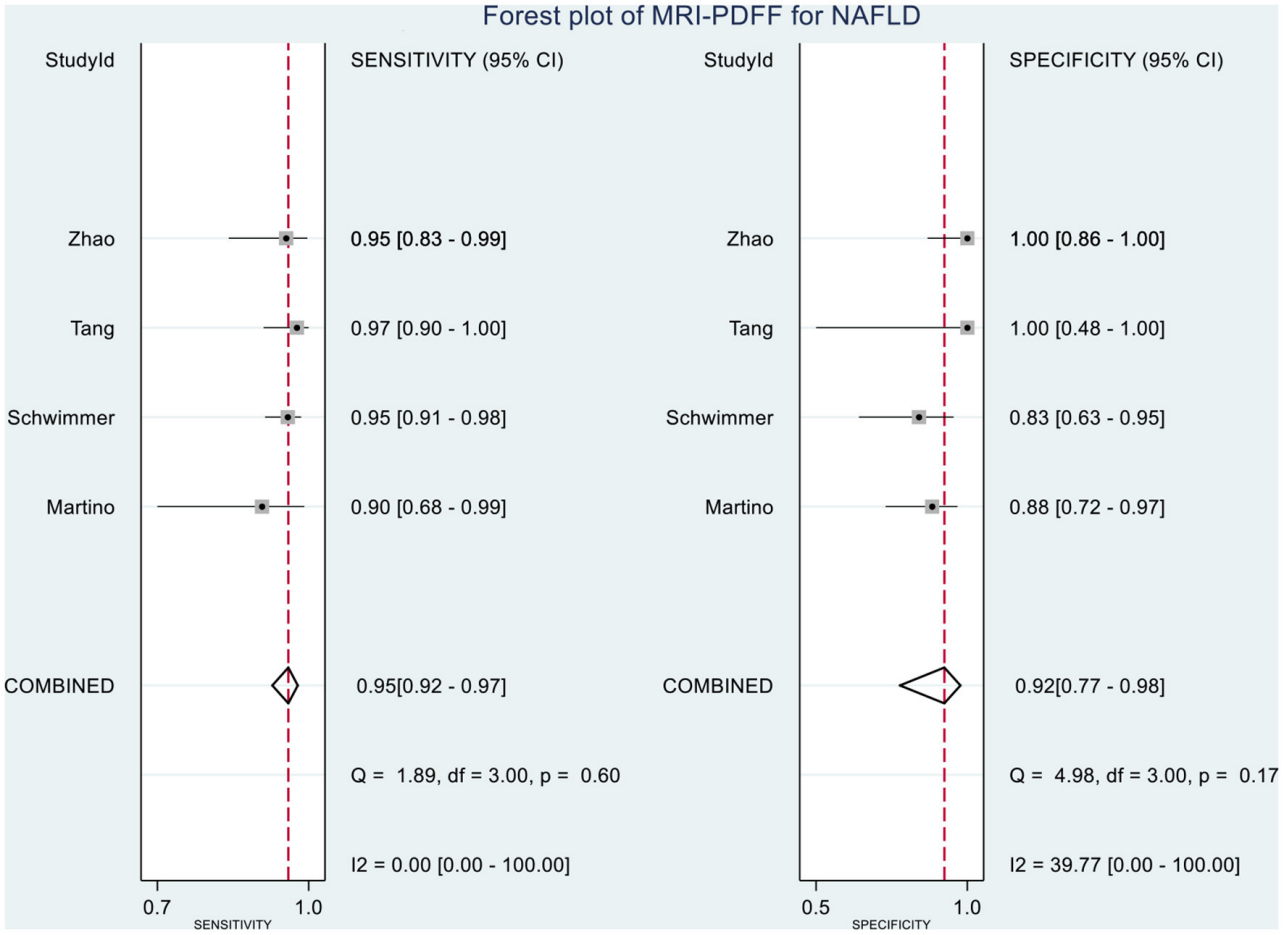
Forest plot to evaluate the sensitivity and specificity of MRI-PDFF in diagnosing NAFLD (S0 vs. S1–S3).

**Figure 9:**
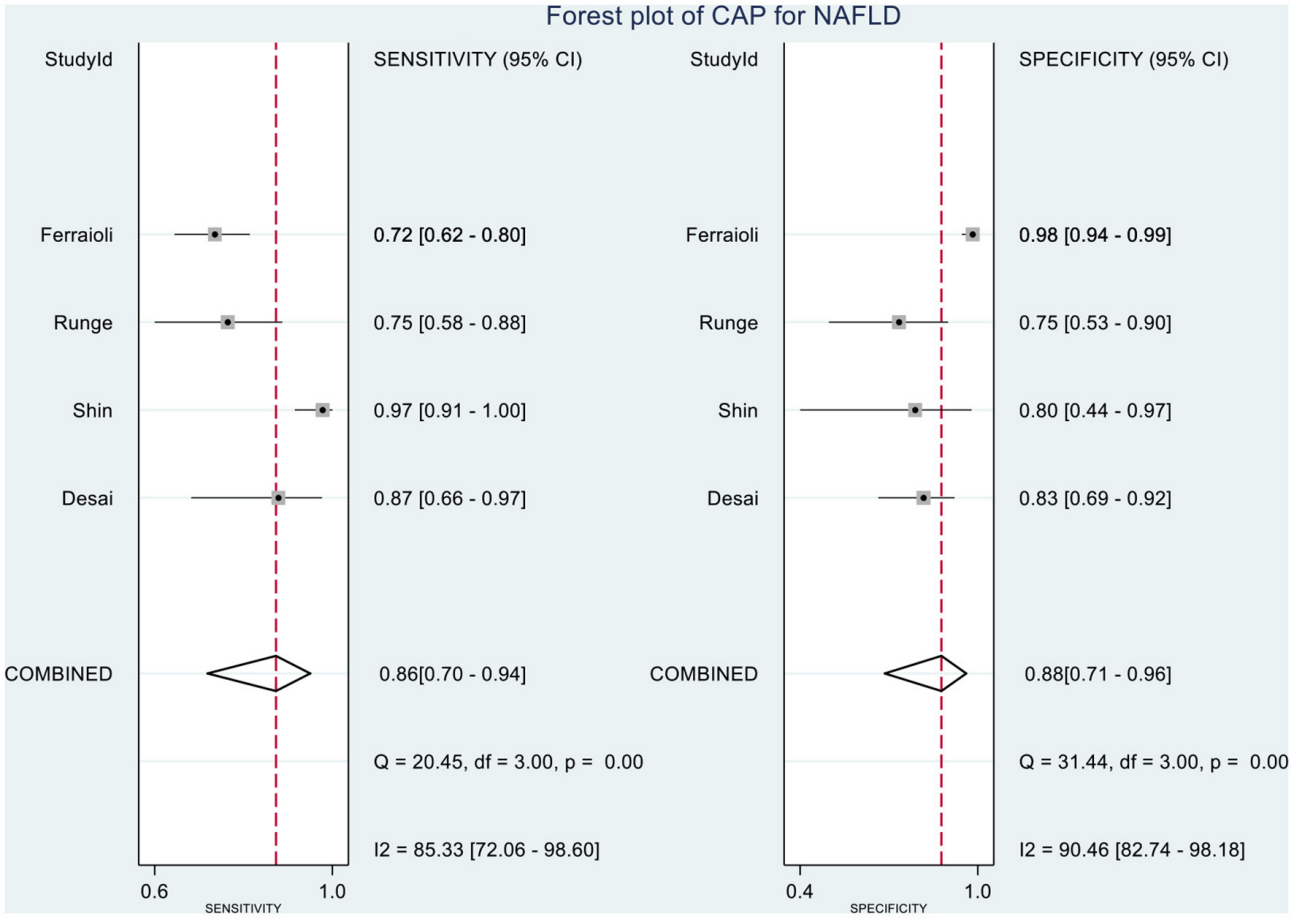
Forest plot to evaluate the sensitivity and specificity of CAP in diagnosing NAFLD (S0 vs. S1–S3).

Deeks' funnel plot asymmetry test showed that MRI-PDFF (*p* = 0.88 > 0.05), TE-CAP (*p* = 0.44 > 0.05), and publication bias were not present among the articles included in S1–3 (Figures 6, 7). A sensitivity analysis was also performed to identify the impact of excluding the articles with the greatest weight on the overall results. This analysis was influenced by the differences in the study population. Overall, there was no significant difference between the odds and residual ratios (Figures 8, 9).

## Discussions and Conclusion

Chronic HS in children and adolescents ( ≤ 18 years) that is not secondary to genetic/metabolic disorders, infections, ethanol consumption, or malnutrition is considered pediatric NAFLD. In children, NAFLD is mostly associated with insulin resistance, central or generalized obesity, and dyslipidemia characterized by high triglyceride and low high-density lipoprotein cholesterol levels (7). NAFLD is the prevalent cause of chronic liver disease worldwide (28, 29). The rising cases of obesity in children and adolescents in recent years have increased the NAFLD cases (18). Although simple HS is generally considered the benign stage in the progression of NAFLD, relevant studies have shown the clinical importance of HS in the progression of NAFLD. Although HS can progress to non-alcoholic steatohepatitis (NASH), it remains broadly underdiagnosed and thus often progresses to liver fibrosis, cirrhosis, and even hepatocellular carcinoma (30, 31). McPherson demonstrated that hepatic lipid deposition directly promotes systemic insulin resistance while leading to metabolic syndrome (32). Therefore, early detection of HS is critical for diagnosing NAFLD (33).

Liver biopsy is invasive, can result in severe complications, and is limited by sampling error and other potential negative outcomes (9). However, it remains the best method for diagnosing and staging non-alcoholic steatohepatitis and liver fibrosis in adults (systematically reviewed by Rinella) (34). The diagnostic guidelines of NAFLD differ significantly in adults and children. The guidelines in children state that liver biopsy continues to be the gold standard for NAFLD diagnosis and typing (7). However, liver biopsy is an invasive method considered only when there is no definite diagnosis by the routine examination and diagnostic approaches and when there is an increased risk of NASH and/or advanced liver fibrosis. All the other versions of liver biopsy guidelines have consistent notions (2, 35). Currently, the best screening method for NAFLD in children, as noted by NASPGHAN, is the evaluation of serum alanine aminotransferase level. However, this method has great limitations, and the clinical manifestations should be comprehensively evaluated. Although ultrasonography serves as the main method to examine NAFLD patients, its accuracy and specificity are not robust (when the liver fat content is <20%, the diagnostic sensitivity of ultrasonography reaches only 55%) (33). Furthermore, NASPGHAN does not recommend ultrasound as a method for detecting HS in children and adolescents. Due to the risk of radiation exposure, and low sensitivity and specificity, the respective edition of the guidelines does not recommend CT as the quantitative detection method for steatosis in children and adolescents (2, 35). The role of MRI-PDFF in detecting HS has been affirmed by the American Association for the Study on Liver Diseases (AASLD) guidelines established in 2017 (2). As highlighted by the previous guidelines, methods (e.g., TE) can be adopted to judge the condition of the pediatric patient to reduce the number of liver biopsies (35).

In the last few years, there have been few articles on the diagnosis of pediatric HS by using MRI-PDFF or TE-CAP (13). Therefore, the diagnostic values of the two methods should be determined urgently. Single-center articles have compared the diagnostic performance of MRI-PDFF with that of TE-CAP in children and adolescents with NAFLD, but no relevant meta-analysis has been conducted (22). However, our meta-analysis included eight original articles (847 participants) with sufficient data for investigating the performances of TE-CAP and MRI-PDFF in diagnosing HS.

This study demonstrated that MRI-PDFF is important for TE-CAP in diagnosing no or S1 steatosis (mild steatosis). Compared with TE-CAP, MRI-PDFF has higher sensitivity and specificity in the preliminary screening of NAFLD. With the pre-test probability of 50%, the Fagan plot analysis demonstrated that MRI-PDFF and TE-CAP have 92% and 88% probability of correctly detecting S1–3 following a “positive” result, respectively. Moreover, the diagnosis would be wrong in only 5% and 13% of the patients with a “negative” measurement of MRI-PDFF and TE-CAP, respectively. In consistence with the previous articles in adults, this study showed that MRI-PDFF has high accuracy in detecting steatosis of NAFLD (36).

In following up patients, MRI-PDFF is suggested to be a better choice than liver biopsy because of the invasiveness of the latter technique. MRI-PDFF has been shown to be a highly precise, accurate, and reproducible non-invasive method for the quantification of liver fat content (33, 34). Furthermore, it has been proven to correlate well with MRS (*r*^2^ = 0.99, *p* < 0.001) (37) and liver biopsy (36). In addition, MRI-PDFF has been demonstrated to be superior to ultrasound, CT, and TE-CAP in the quantification of liver fat content (13, 37). Similar results have been obtained in previous studies. These observations further prove the value of MRI-PDFF in the diagnosis of NAFLD.

TE-CAP is another novel technology that has been reported to accurately measure HS severity in several studies conducted in different adult patients and children (37). However, the suitability of TE-CAP for use in children and reliable indicators for its application remain to be clarified because fewer studies have been conducted in children than in adults (38, 39). In a meta-analysis conducted by Gu et al. (36), TE-CAP exhibited high performance for the staging of HS in NAFLD. It has been reported that TE-CAP is less influenced by sampling errors than liver biopsies, as TE-CAP explores a liver volume approximately 100 times larger than liver biopsies. In addition, TE-CAP is designed to specifically target the liver, with a relatively easy operation, and it can provide immediate results. Additionally, it can be inexpensively integrated into FibroScan. Moreover, TE-CAP can be simultaneously performed with measurements of liver stiffness in the identical liver volume. Thereby, both fibrosis and steatosis can be simultaneously evaluated, consequently facilitating the investigation and follow-up of NAFLD patients.

Fe et al. have found that CAP has better sensitivity and specificity than ultrasound in the diagnosis of non-alcoholic fatty liver in children (9). Furthermore, Shin et al. have reported that CAP can differentiate between the presence and absence of HS in pediatric NAFLD patients by using a cutoff value of 241 dB/m with a sensitivity of 98.7% and specificity of 80.0% (29). However, CAP is limited in evaluating steatosis grades, especially in children with BMI >30 kg/m^2^. In the articles included in this study, CAP was repeatedly proven to be consistent with liver biopsy and magnetic resonance quantitative analysis of HS in diagnosing NAFLD of children (18, 19). In a previous study, CAP has been proven to correlate well with liver biopsy (*r*^2^ = 0.90, *p* < 0.001), with 0.87 sensitivity, 0.83 specificity, positive predictive value of 0.71, negative predictive value of 0.93, and area under the curve value of 0.93 (95% CI, 0.87–0.99) for predicting steatosis (19).

This study had some limitations. First, the MRI examinations in our study were performed using a 1.5- or 3.0-T MR imager, although a recent study has shown that there is no difference in terms of the calculated MRI-PDFF between the two imagers (40). Second, concerning the geographic regions where TE-CAP and MRI-PDFF were performed, the cutoff values used by the individual centers may contribute to inconsistencies in TE-CAP data. Furthermore, multicenter and large cohort studies or large population-based studies are also needed to validate the clinical application of TE-CAP and MRI-PDFF in diagnosing HS.

In summary, both of the two methods showed excellent diagnostic performances for NAFLD in children and adolescents, and the accuracy and specificity of MRI-PDFF were more prominent than those of TE-CAP.

## Data Availability Statement

The original contributions presented in the study are included in the article/supplementary material, further inquiries can be directed to the corresponding authors.

## Author Contributions

SJ and JZ: conceptualization and funding acquisition. YZ and JL: data collection. XG and MC: data analysis. SJ, SZ, and JZ: investigation, writing original draft, and writing—review and editing. JZ: project administration. All authors have read and approved the manuscript.

## Funding

This work was supported by the Science Technology and Innovation Committee of Shenzhen (2021N062-JCYJ20210324115408023).

## Conflict of Interest

The authors declare that the research was conducted in the absence of any commercial or financial relationships that could be construed as a potential conflict of interest.

## Publisher's Note

All claims expressed in this article are solely those of the authors and do not necessarily represent those of their affiliated organizations, or those of the publisher, the editors and the reviewers. Any product that may be evaluated in this article, or claim that may be made by its manufacturer, is not guaranteed or endorsed by the publisher.
